# Speech Intonation Induces Enhanced Face Perception in Infants

**DOI:** 10.1038/s41598-020-60074-7

**Published:** 2020-02-21

**Authors:** Louah Sirri, Szilvia Linnert, Vincent Reid, Eugenio Parise

**Affiliations:** 1 Department of Education, Manchester Metropolitan University, Manchester, UK; 2 Department of Psychology, Lancaster University, Lancaster, UK; 3 School of Psychology, University of Waikato, Hamilton, New Zealand

**Keywords:** Social behaviour, Human behaviour

## Abstract

Infants’ preference for faces with direct compared to averted eye gaze, and for infant-directed over adult-directed speech, reflects early sensitivity to social communication. Here, we studied whether infant-directed speech (IDS), could affect the processing of a face with direct gaze in 4-month-olds. In a new ERP paradigm, the word ‘*hello*’ was uttered either in IDS or adult-direct speech (ADS) followed by an upright or inverted face. We show that the face-specific N290 ERP component was larger when faces were preceded by IDS relative to ADS. Crucially, this effect is specific to upright faces, whereas inverted faces preceded by IDS elicited larger attention-related P1 and Nc. These results suggest that IDS generates communicative expectations in infants. When such expectations are met by a following social stimulus – an upright face – infants are already prepared to process it. When the stimulus is a non-social one –inverted face – IDS merely increases general attention.

## Introduction

Human infants demonstrate a strong early sensitivity to a range of social stimuli including faces, eyes and speech, all of which are attractive and interesting to them. For instance, both newborns and young infants orient toward and look longer at human faces compared to other equally complex stimuli^1–4^. Face inversion disrupts such preference^5^, suggesting the existence of a dedicated mechanism specifically tuned to upright, but not inverted, face-like patterns^4,6^.

Newborns also prefer to look at faces with open compared to closed eyes^7^, and to faces with direct compared to averted eye gaze^8,9^. Direct gaze can also facilitate the face processing itself, helping infants to encode and recognize specific individual faces^10^. At 4 months, infants show a larger N290 component to faces with direct gaze contrasted to faces with averted gaze^8^. The N290 is an infant event-related brain potential (ERP), a precursor of the adult N170 component^11,12^ known to reflect face perception^13,14^. In adults, face inversion disrupts configural-holistic face processing^15^, and similarly, face inversion disrupts the N290 effect for direct gaze in infants^16^.

Sensitivity to social stimuli is not limited to faces and direct gaze. Newborns and infants also differentiate^17^ and prefer^18,19^ infant-directed speech (IDS) compared to adult directed speech (ADS). Relative to ADS, IDS has higher and more variable pitch, slower speed, longer pauses, limited vocabulary, shorter utterances and vowel alterations^18,20,21^. Newborns and 1-month-old infants look longer at a face when it produces IDS compared to ADS^22^. Robust preference for IDS over ADS does not depend on the gender of the speaker^19^ or the native language of the infant^23^, suggesting that IDS has a universal appeal. ERP studies have also shown that IDS enhances the acoustic processing (200–400 ms time window) of speech^24,25^ and boosts the neural activity and arousal (600–800 ms time window), particularly in response to familiar words^24^.

Several hypotheses have been proposed to account for infants’ fascination with social stimuli such as IDS, faces and eyes^26^. Amongst others, it has been proposed that infants interpret these stimuli as ostensive communicative signals^27,28^. These signals demonstrate the communicative intentions of someone, clarifying who is the addressee of the communication^28,29^. Infants prefer faces for they represent a main source of social information^30^. Specifically, direct eye contact can serve as an ostensive signal suggesting to the infants that they are communicatively addressed. Just like direct gaze, IDS might indicate to the infant that they are the addressee of the communication^28^. Notably, direct gaze and IDS show some ERP similarities when compared to their non-ostensive counterparts^31^. Parise and Csibra^31^ showed that at 5 months, an early latency ERP deflection at the centre of the scalp was more pronounced in response to direct relative to averted gaze, and to IDS relative to ADS, suggesting a common neural representation and, possibly, a common interpretation. However, whether infants prefer ostensive signals because they are simply more arousing^32^, or because they attract a communicative interpretation, is an open question.

Here, we were asking whether face perception in infants, indexed by the N290 component, could be modulated by a preceding social signal such as IDS. Investigating the effect of speech intonation on the infant N290 component to faces, we specifically aimed to reproduce that same N290 effect already found, in infants^8^ and adults^33^, with manipulation of the gaze direction. We reasoned that infants interpret faces with direct eye contact as potential sources of communication^30^. Previously, however, the communicative signal (eye contact) and its source were presented at the same time, embedded into the same visual stimulus^8^, making it self-evident that the face was the source of the communication. In this study, we dissociated the communicative signal from its source, both in time and in perceptual modality. We developed a novel electroencephalographic (EEG) paradigm in which we used IDS as an auditory communicative prime, followed by the presentation of a face. If IDS suggests the presence of a communicative source, following the speech face processing will be enhanced, even though the communicative signal (IDS) and its source (the face) are presented sequentially rather than simultaneously, and belong to different sensorial domains.

With this hypothesis, we studied the effect of IDS on face perception in 4-month-old infants using ERPs, where an ERP effect is a statistically significant difference in amplitude between the two conditions, ADS vs. IDS, in a given time window. In Experiment 1, while their EEG was recorded, infants heard the word ‘*hello*’ uttered in either ADS or IDS intonation. Following the prime word, a static face with direct gaze appeared on the presentation screen. Experiment 2 followed the same procedure, except that faces were inverted. This design kept the visual stimulus constant within each experiment, allowing the infants to form an expectation of what they see, and allowing us to study the influence of IDS on infants’ visual expectation of faces. Just like in previous studies^24,25,31^, we expect that relative to ADS, IDS elicits larger auditory ERPs, in the 200–400 ms and 600–800 ms time windows, indicating that infants process the two intonations differently. Our main prediction is that IDS enhances the perception of upright faces: upright - but not inverted faces - would elicit a larger N290 when preceded by IDS compared to ADS. This would be reflected by larger N290 amplitudes in the IDS compared to the ADS condition. On the other hand, as it has also been proposed^32^, if ostensive signals simply enhance attention and do not generate social expectations, the visual Nc component (related to allocation of attention^34,35^) will be larger for stimuli following IDS compared to ADS in both experiments, regardless of faces orientation.

## Results

### Effect of IDS on upright faces - experiment 1

The first experiment aimed at measuring the effect of speech intonation on face processing. We presented 4-month-old infants with the word “*hello*”, lasting 580 or 720 ms respectively. After an inter stimulus interval varying randomly between 200 to 400 ms, the utterance was followed by the presentation of an upright female face on a computer screen for 1000 ms. We measured both the ERPs to the speech and to the faces.

#### Auditory ERPs

**Figure 1:**
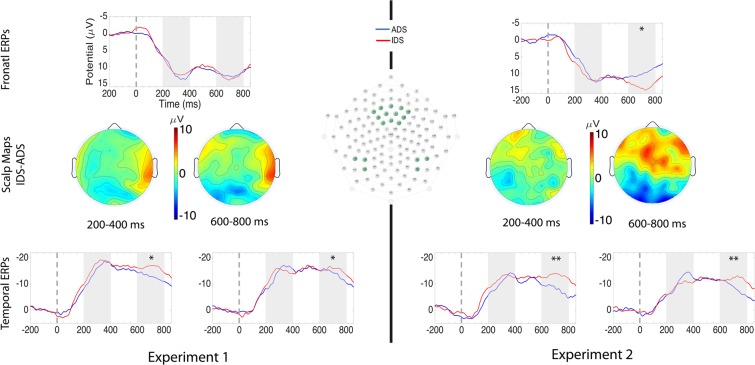
Auditory ERPs in Experiment 1 and Experiment 2. In the center, selected electrodes and scalp distributions of the IDS-ADS differences. The grand-average waveforms are shown in the upper panels for the frontal recording site and in the bottom panels for the temporal recording sites. Negative is plotted up and time 0 is the onset of the auditory stimulus. Grey shadings indicate the analyzed time windows (200–400 ms and 600–800 ms); statistical significance is indicated through one (*p* < 0.05) or two (*p* < 0.01) stars. Topographical maps in the middle show scalp distribution of ERP differences in the 200–400 ms and 600–800 ms time windows. Darker colors reflect greater amplitudes.

Auditory ERPs are shown on Fig. 1 (left). We looked at two ERP time windows in response to speech stimuli: the deflections from 200 to 400 ms and from 600 to 800 ms, both measured over the fronto-central and temporal recording regions. On the fronto-central region we ran a *t*-test with Speech Type (IDS *vs*. ADS); on temporal regions we ran an ANOVA with Speech Type (IDS *vs*. ADS) and Hemisphere (left *vs*. right) as within-subject factors. In the 200–400 ms time window, there was no main effect of Speech Type or interactions with this factor, either on frontal-central or on the temporal sites (all *p*s > 0.35).

The amplitudes in the 600–800 ms time window did not differ between ADS and IDS over the frontal recording region (*t*(17) = −0.38; *p* = 0.71), there was, however, a main effect of Speech Type over temporal electrodes (*F*(1,17) = 6.88; *p* = 0.02; *η*^2^_*p*_ = 0.29) with more negative amplitudes for IDS compared to ADS. Thirteen out of 18 infants showed this effect (Wilcoxon’s *Z* = 2.20, *p* = 0.03).

#### Visual ERPs

**Figure 2:**
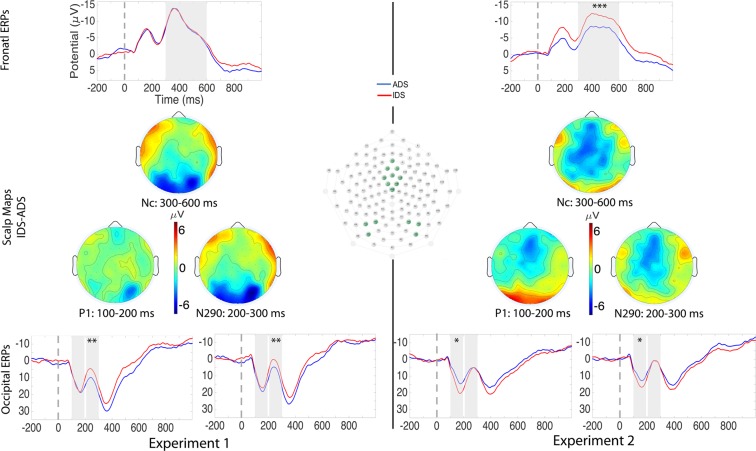
Visual ERPs in Experiment 1 and Experiment 2. In the center, selected electrodes and scalp distributions of the differences for upright (left) and inverted (right) faces. The grand-average waveforms are shown in the upper panels for the fronto-central recording site and in the bottom panels for the occipital recording sites. Negative is plotted up and time 0 is the onset of the visual stimulus. Grey shadings indicate the analyzed time windows of the visual ERPs: P1 (100–200 ms), N290 (200–300 ms) and Nc (300–600 ms); statistical significance is indicated through one (*p* < 0.05), two (*p* < 0.01) or three (*p* < 0.001) stars. Topographical maps in the middle show scalp distribution of ERP differences for the Nc (top), P1 (bottom left) and N290 (bottom right). Darker colors reflect greater amplitudes.

Visual ERPs are shown on Fig. 2 (left). We looked at three ERP components in response to faces: the early perceptual components P1 (100 to 200 ms) and N290 (200 to 300 ms) over occipital regions, and the middle latency attention related Nc component (300 to 600 ms) over the fronto-central region. On occipital regions, we conducted an ANOVA with Speech Type (IDS *vs*. ADS) and Hemisphere (left *vs*. right) as within-subject factors; on the fronto-central region we ran *t*-tests with Speech Type (IDS *vs*. ADS) as within-subject factor. The P1 did not reveal any significant main effect or interaction (all *p*s > 0.20). The N290 revealed main effects of Speech Type (*F*(1,18) = 12.43; *p* = 0.002; *η*^2^_*p*_ = 0.41) and Hemisphere (*F*(1,18) = 9.18; *p* = 0.007; *η*^2^_*p*_ = 0.34). The N290 amplitudes for upright faces were more negative following IDS than following ADS intonation. Fourteen out of 19 infants showed this effect (Wilcoxon’s *Z* = 2.70, *p* = 0.007). We did not find a significant interaction of Speech Type × Hemisphere (*p* = 0.70).

The Nc amplitudes did not reveal an effect of Speech Type (*t*(18) = 0.15; *p* = 0.88). Further analyses of visual ERPs are reported in the Supplementary Information.

These results are in line with our prediction. IDS elicited larger auditory responses than ADS and the face-specific N290 component was larger to faces preceded by IDS. Furthermore, the Nc to faces was not influenced by speech intonation. As the Nc reflects general attentional processes^33,34^, this result suggests that IDS does not merely increase attention to faces. Rather, it has a specific effect on the early perceptual processing stages of faces. However, we cannot exclude the possibility that IDS enhances the early visual perception of any following stimulus. In Experiment 2 we addressed this possibility by presenting inverted faces.

### Effect of IDS on inverted faces - experiment 2

We used the same paradigm employed in Experiment 1 to investigate whether IDS enhances early perceptual processing of any following visual stimulus. Presenting inverted faces disrupts face-specific configural-holistic processes indexed by the N170/N290 both in infants^35^ and adults^15^. We reasoned that if IDS specifically affects the early processing of upright faces, the N290 in response to inverted faces will not be modulated by speech intonation. If, on the other hand, IDS evenly affects the processing of any type of visual stimulus, the pattern of results of the P1, the N290 and the Nc in this experiment will be similar to that found in Experiment 1.

#### Auditory ERPs

Auditory ERPs are shown on Fig. 1 (right). There was no effect of Speech Type in the 200–400 ms time window either over the frontal (*t*(17) = −1.03; *p* = 0.32) or the temporal areas (*F*(1,17) = 0.01; *p* = 0.94; *η*^2^_*p*_ < 0.01).

In the 600–800 ms time windows, we found a significant effect of Speech Type over the frontal area (*t*(17) = −2.50; *p* = 0.02), with 15 out of 18 infants showing the effect (Wilcoxon’s *Z* = 2.20; *p* = 0.03). Similarly, we found a significant effect of Speech Type over the temporal areas (*F*(1,17) = 9.84; *p* = 0.006; *η*^2^_*p*_ = 0.37); 13 out of 18 infants showed the effect (Wilcoxon’s *Z* = 2.55; *p* = 0.01).

#### Visual ERPs

Visual ERPs are shown on Fig. 2 (right). There was a main effect of Speech type on the P1 component (*F*(1,17) = 5.85; *p* = 0.03; *η*^2^_*p*_ = 0.26), with more positive amplitudes of IDS compared to the ADS condition. Eleven out of 18 infants showed this effect (Wilcoxon’s *Z* = 1.81; *p* = 0.07). There was no main effect of Hemisphere, or interaction of Speech Type × Hemisphere (all *p*s > 0.28).

As predicted, we found no significant main effect of Speech Type on the N290 component (*F*(1,17) = 0.58; *p* = 0.46; *η*^2^_*p*_ = 0.03). The main effect of Hemisphere was marginally significant (*F*(1,17) = 4.20; *p* = 0.06; *η*^2^_*p*_ = 0.20) The interaction between Speech Type and Hemisphere was not significant (*p* > 0.47).

We found a significant effect of Speech Type on the Nc component (*t*(17) = 4.29; *p* < 0.001), with more negative amplitudes for inverted faces following IDS compared to ADS. Fifteen out of 18 infants showed this effect (Wilcoxon’s *Z* = 3.03; *p* = 0.002).

### Cross-experiment comparison**s**

We directly compared the two experiments with Experiment (Exp.1 vs. Exp.2) as additional between-subjects factor.

#### Auditory ERPs

In the 200–400 ms time windows we found no significant interaction Experiment × Speech Type, over the frontal (all *p*s > 0.30) or temporal areas (all *p*s > 0.12).

In the 600–800 ms time windows, over the frontal area, we found a tendency for the interaction Experiment × Speech Type (*F*(1,34) = 3.60; *p* = 0.07; *η*^2^_*p*_ = 0.10). Over temporal areas there was no interaction with the factor Experiment (*p*s > 0.31).

#### Visual ERPs

In response to visual stimuli, we found three interactions Experiment × Speech Type on the three ERP components.

On the P1 component (*F*(1,35) = 7.66; *p* = 0.009; *η*^2^_*p*_ = 0.18), responses to stimuli preceded by IDS were larger than those preceded by ADS, but only in Experiment 2 with inverted faces.

As expected, the interaction on the N290 component (*F*(1,35) = 8.32; *p* = 0.007; *η*^2^_*p*_ = 0.19) further confirmed that only upright faces (Experiment 1) elicited greater N290 amplitudes when preceded by IDS relative to ADS.

For the Nc component we found the same pattern of results observed on the P1 (*F*(1,35) = 5.09; *p* = 0.03; *η*^2^_*p*_ = 0.13), with the Nc effect only present in Experiment 2 with inverted faces.

The P1 effect in Experiment 2 was not predicted. To investigate whether it was similar to the N290 effect observed in Experiment 1, or related to attention like the Nc effect in Experiment 2, for every infant we subtracted ADS from IDS on each of the three ERP components, then we ran pairwise correlations of these three differences: between P1 and Nc, between N290 and Nc, and between P1 and N290 (see Fig. S1 in the Supplementary Information). We included all participants from both experiments. To account for the difference in polarity between P1 (positive) and N290/Nc (negative), we transformed all the P1 difference values into their corresponding negatives. We found a positive correlation for the IDS-ADS difference between P1 and Nc (*r* = 0.52, *p* < 0.01), and a negative correlation between N290 and Nc (*r* = −0.54, *p* < 0.01). Finally, we found a negative correlation for the IDS-ADS difference between P1 and N290 (*r* = −0.87, *p* < 0.01). Conducting the same correlations for the two experiments separately led to the same pattern of results. The positive correlation between P1 and Nc suggests similar underlying attentional mechanisms, whereas the negative correlations involving the N290 suggest, for this component, a different mechanism.

The results of Experiment 2 show that IDS influenced the early perceptual processing of inverted faces as reflected by the P1 component. Furthermore, IDS caused an increase of attention allocated to inverted faces, as reflected by the Nc component. Together with the comparisons across experiments and the correlations between IDS-ADS differences on the ERP components, these results support our main hypothesis that IDS selectively enhances face perception and this process cannot be reduced to a generalized increase of attention.

This pattern of results indicates distinct cognitive mechanisms depending on what the infants saw after hearing IDS: a potential source of the communicative signal (upright face) or a configuration (inverted face) that they have hardly encountered in their everyday life social interactions.

## Discussion

In order to study whether infants attribute the status of communicative source to faces, we developed a new paradigm. We dissociated, in time and sensorial modality, a communicative signal from its source, then we measured the infant face sensitive N290 ERP component. We expected to find an effect of speech intonation on the N290 to faces, akin to that observed in previous studies using faces with direct vs. averted gaze^8^. As expected, we found such effect: IDS selectively enhanced face perception in 4-month-old infants. In our study, this effect could only be caused by the intonation of the utterance preceding the faces, given that across experimental conditions we used the same photographs of faces, all portraying direct gaze. This finding supports the hypothesis that when ostensively addressed, infants look for the source of social communication when that is not immediately evident. The effect was only present for upright, but not for inverted faces. For inverted faces, however, we found differences on the visual P1 and Nc components, with larger amplitudes for inverted faces preceded by IDS. In both experiments, we found that compared to ADS, IDS elicited greater amplitudes in the 600–800 ms time window on temporal areas^23,31^. However, on the same time window in frontal areas, we found that IDS elicited larger amplitudes than ADS in Experiment 2. This result was unexpected and deserves further investigation.

Our results have a number of implications for the understanding of development and communication. First, we successfully increased the N290 amplitude to faces by using a communicative signal other than direct gaze, replicating previous results in infants (with pictures of the face; 8) and in adults (with live faces^36^). This excludes the possibility that such N290 effect is due to the low-level characteristics of the visual stimulus. Rather, in line with our theoretical premises^27,30^, we believe that infants interpret both direct gaze and IDS as communication directed to them and consequently, they dedicate more neural resources to early stages of face processing. Crucially, just like previously shown with direct eye gaze^16^, IDS did not produce such effect on inverted faces. We propose that both direct gaze^8^ and IDS provide the infant with the feeling of being addressed by a conspecific^28^. Enhanced face perception could stem from such feeling, either induced by eye contact or by IDS. Our study design facilitated infants’ visual expectations: they could accurately predict what they will see after the speech, and whether the visual stimulus would be a suitable communicative source. IDS in Experiment 1 effectively primed the infant visual system to respond to faces, leading to the predicted N290 effect.

Second, we observed the N290 effect in a sequential paradigm, where the communicative signal was presented prior to its source, preventing the participants from immediately spotting the source of the communication. Nevertheless, our result matched the findings of previous studies^8,36^ where the source of the signal was self-evident. Assuming a common cause behind these N290 matching results, we propose that infants are not interested in communicative signals per se, but are rather interested in finding communicative partners^31^. Here, when an upright face was presented, infants interpreted it as the source of IDS. Their responses to upright faces notably differed from those to inverted faces. This is a result that might be driven and shaped by experience of communicative interactions, where infants more often encounter upright than inverted faces. However, infants encounter many adult faces who often address them using IDS. It is possible that infants have just established a simple association between IDS and faces, very often co-occurring. Though we cannot completely rule out this alternative interpretation, we believe that familiarity on its own cannot fully explain our findings, as infants also have considerable experience with adult faces using ADS. Moreover, if the association between IDS and face was stronger than that between ADS and face, an Nc effect in Experiment 1 would probably have been present, which was not the case.

Third, these results speak about distinct processing mechanisms and domain specific processes. An alternative explanation of our findings is that all the observed effects in the visual ERPs, including the N290 effect to upright faces, are different signatures of the same attentional mechanism. It has been recently suggested that attention plays a role in the generation of the infant N290^37^. It is possible to speculate that IDS increases infants’ attention to the following visual stimulus independently of its nature. We found attention as driving mechanism on its own not fully convincing for several reasons. The difference between the two experiments at the early processing stages - as indexed by the P1 and N290 components - speaks against one common process. Though both the P1^38^ and the N290^37,39^ have been found in studies on infants’ attention, in our study the stimuli between the two experiments are matched pixel by pixel but differ in their psychological meaning: human (upright) faces are special stimuli from at least birth^3,40^. Our results suggest that the P1 and N290 are neural correlates of different processes, rather than just different aspects of the same (attentional) process, with the N290 being a well-studied correlate of face perception in infants. The middle latency ERP differences between the two experiments support our argument. The visual Nc component, well known to reflect infants’ attention^33,34,41^, showed a difference between conditions only in reaction to inverted faces in Experiment 2, indicating that speech intonation caused a difference in allocation of attention to inverted but not to upright faces.

One might ask why infants do not dedicate more attentional resources to upright faces followed by IDS once they have classified them as communicative sources, that is: why there is no Nc effect in Experiment 1? A simple possibility is that, because all upright faces in our study exhibited direct gaze, all faces deserved an equal amount of attention. An alternative explanation is the following. According to the theory^27–29^, one aspect of the comprehension of communication is the recognition of a communicative intention. The other aspect involves understanding the informative intention, that is the referential extension to the content of the communication. In our study, upright faces did not provide the infants with any referential signal. Infants could have been waiting for further communicative signals from upright faces (e.g. referential gaze) for their attentional resources to be directed toward a referent. Notice that also^31^ did not find differences on the Nc in response to individual ostensive signals or combinations of them, whereas some studies found enhanced Nc to objects following ostensive signals^42,43^. Hence, within this framework, the absence of an Nc effect in Experiment 1 is not surprising.

Finally, the positive correlation of the differences IDS-ADS between P1 and Nc, the negative correlation between N290 and Nc, and the negative correlation between P1 and N290 are not compatible with a domain general attentional account. Our results suggest, at least to some extent, that the larger the effect of speech on the N290, the smaller the effect is on the attention related Nc. Rather, we propose that IDS taps onto different mechanism in the two experiments. In Experiment 1, infants are prepared to see a face and all their cognitive resources are dedicated to encode the source of the social communicative signal. Note that, in our study, the latency of the posterior N290 closely matches the latency of the detection of ostension on central scalp areas that was observed earlier^31^, where the source of the communication was clear. This suggests that infants look for and recognize a source of communication during the early stages of stimulus processing.

Inverted faces are unlikely to be the source of social signals in the experience of 4-month-olds. If infants cannot dedicate more perceptual resources to specifically encode a recognizable source of communication, they can still use their attentional resources to process what is presented on the screen, as reflected by the Nc effect in Experiment 2. In 5-month-olds, similar Nc effects have been observed with different communicative signals, such as the infant’s own name^42^ and direct eye contact^43^, preceding the on-screen presentation of objects. There is evidence^44–46^ that infants consider inverted faces just like objects, categorically different from upright faces.

It is difficult to adopt a lean interpretation when looking at this pattern of results. If speech intonation, and social communication in general, would merely enhance attention in infants^32^, we would have observed similar effects for both upright and inverted faces. Instead, we observed two distinct cognitive processes following the same signal. This suggests that 4-month-olds used IDS differently in the two experiments: to look at the source of communication (enhanced face perception) in one case, and to orient their attention to the external world in the other case. In line with previous research on early social communication, we conclude that infants have rich representations of communicative signals, such as direct gaze and IDS, that they flexibly use to guide their cognitive processes in response to the actual social context of any information that is offered them.

## Methods

### Participants

In Experiment 1, thirty-five infants took part in the study: 18 infants (mean age: 144.78 days; range: 115 to 177 days; 5 female) contributed to the auditory ERP analysis, and 19 infants (mean age: 146.47 days; range: 115 to 177 days; 5 female) contributed to the visual ERP analysis. In Experiment 2, thirty-one infants took part in the study: 18 infants contributed to the auditory ERP analysis (mean age: 135.61 days; range: 117 to 161 days; 5 female) and 18 infants contributed to the visual ERP analysis (mean age: 136.06 days; range: 117 to 162 days; 3 female). In both experiments the majority of the infants were included in both auditory and visual ERP analysis (Experiment 1: *n* = 16, Experiment 2: *n* = 16; see Supplemental Information for analyses on these subsets of participants). However, some infants contributed enough artifact free segments only in the auditory (Experiment 1: *n* = 2, Experiment 2: *n* = 2) or only in the visual (Experiment 1: *n* = 3, Experiment 2: *n* = 2) condition. All additional participants were not included in the statistical analyses due to an insufficient amount of artifact free trials or technical issues.

All infants were born healthy (≤37 weeks of gestation), and were recruited from a database of parents from the local area who expressed an interest in taking part in developmental research studies. Parents were informed about the aim of the study and gave informed written consent before participation. Infants received a book for their participation. The study was conducted in conformity with the declaration of Helsinki and approved by the University Research Ethics Committee, at Lancaster University.

### Stimuli

In both experiments, the auditory stimuli were the same as in^47^, shared by the senior author: the greeting word “*hello*” uttered by a female voice in either IDS or ADS. Audio files were digitized and edited with Adobe Audition (CS 5.5), at 16-bit resolution and 44 kHz sampling rate. The speech had different length, 580 ms for ADS and 720 ms for IDS, but primarily differed in pitch and intensity. The mean intensity of speech was 75 dB for ADS and 85 dB for IDS. Auditory stimuli were delivered through loudspeakers located behind the monitor.

Visual stimuli consisted of 9 color photographs with a white background, portraying white female adult faces with a neutral expression selected from the NimStim repository^48^. The authors^48^ shared the visual stimuli, including instructions as to which faces from their repository can be used in our study and for publication. Each picture measured 355 ×x 473 pixels. At the viewing distance of 60 cm from a 19-inch CRT monitor, each picture subtended horizontal and vertical visual angle of 16.1° and 21.7°, respectively. In Experiment 2 we used the same pictures, but rotated at 180° (examples on Fig. 2).

### Procedure

Infants sat on their parents’ lap throughout the whole experiment. Mothers were instructed not to talk to their infants during the presentation of the stimuli. Each trial consisted of an auditory and a visual stimulus and the experiment consisted of one block including 108 trials, 54 trials in each ADS and IDS condition. All stimuli were presented with Matlab^**®**^ (v. 2014b), using PsychToolBox functions and custom-made scripts. Each trial started with a central dynamic visual attention grabber swirling on a grey background for 2150 ms, after which it froze while the auditory stimulus (“*hello*”) was played. The attention grabber was centred on the screen. Then the attention grabber disappeared, and a face appeared on the screen, with the eyes located in the region previously occupied by the attention grabber. The stimulus onset asynchrony between the auditory and visual stimuli was randomized between 1050 and 1250 ms. The face remained on the screen for 1000 ms. During the inter-trial interval, the grey screen remained blank for a random period varying from 1000 to 1200 ms. To further attract infants’ attention during the experiment, there were 6 different dynamic attention grabbers, changing every 6 trials. The presentation order of the conditions was randomised, and trials were presented as long as the infant was attentive. If the infant lost interest, an animated spiral and a jingle were presented to reorient attention to the presentation screen. If the infant became fussy, the animated spiral was played again or the experimenter gave a short break and played with the baby. The session ended if the infant was no longer attracted to the screen. The whole experiment lasted approximately 15 minutes and was video-recorded for offline data editing purposes.

### EEG recording and analysis

The EEG was continuously recorded using a 124-channel Hydrocel Geodesic Sensor Net, referenced online to the vertex (Cz). The EEG signals were amplified via an EGI NetAmps 400 amplifier, digitized at 500 Hz sampling rate with a 200 Hz lowpass filter. Offline the EEG was filtered between 0.3–30 Hz. The 150 ms of silence at the begin of both audio files were compensated, and EEG signals in response to auditory stimuli were segmented into 1050 ms epochs including 200 ms before stimulus onset and 850 ms post stimulus onset. EEG in response to visual stimuli were segmented into 1200 ms epochs including 200 ms before stimulus onset to 1000 ms post stimulus onset.

Both automatic and manual artifact detection were executed. Bad channels were automatically rejected if the average amplitude of an 80  ms gliding window exceeded ±150 μV. In addition to the automatic artefact detection, each individual epoch was visually inspected and further epochs or channels were included or rejected. Segments containing eye-blinks or eye-movements or more than 13 bad channels (<10% of all electrodes) were rejected. During the visual inspection of the data, EEG segments time locked to the speech were processed first and did not require the infant to attend towards the following visual stimulus. Segments time locked to the face were excluded if the video recording showed that the infants did not attend towards the stimuli. Bad channels of included segments, that is segments with less than 13 bad channels (10%), were replaced using spherical spline interpolation. To compute the ERPs we averaged auditory and visual segments separately, for each participant and each condition, resulting in 2 auditory ERPs (ADS or IDS) and 2 visual ERPs (faces preceded by ADS or IDS). All ERPs were re-referenced to an average reference and baseline corrected to the 200 ms pre-stimulus onset. Previous infant EEG studies have observed reliable and interpretable data in as few as 7 trials per condition^49,50^. Here, the minimum number of artifact free trials for inclusion was 8 per condition, both for the auditory and the visual ERPs. In Experiment 1, the mean number of artifact free trials for auditory ERPs was 18 (ranging from 9 to 39) in ADS and 17 in IDS (ranging from 8 to 31) condition. For the visual ERPs, the mean number of artifact free trials was 17 (ranging from 9 to 36) in ADS and 17 in IDS (ranging from 8 to 32) condition. In Experiment 2, the mean number of artifact free trials for auditory ERPs was 20 (ranging from 8 to 39) for ADS and 20 (ranging from 9 to 37) for IDS. For the visual ERPs the mean number of artifact free trials was 20 (ranging from 8 to 39) for ADS and 21 (ranging from 11 to 41) for IDS.

Based on previous findings^24,31^, we identified the time windows from 200 to 400 ms and 600 to 800 ms for responses to speech stimuli, over both the fronto-central and temporal recording sites. For the fronto-central area, we averaged the mean amplitudes of 12 channels (4, 5, 6, 11, 12, 13, 19, 20, 24, 112, 118 and 124), approximately corresponding to the F3, F4, Fz, C3 and C4 locations in the 10–20 system. For the temporal areas, we averaged the mean amplitudes of 3 channels over the left (58, 59 and 65, corresponding to the T5 area) and over the right (90, 91, 96 corresponding to T6) recording sites (Fig. 1).

For the visual stimuli, we analyzed the P1 (100 to 200 ms), the N290 (200 to 300 ms) and the infant specific Nc component (300 to 600 ms) related to the allocation of attention^33,34^. For the P1 and N290 component we averaged the mean amplitudes over the left (channels: 65, 66, 69) and right (channels: 84, 89, 90) occipital areas^8,16^. For the Nc component, we averaged the mean amplitudes over the frontal-central area (5, 6, 7, 11, 12, 13, 106, 112, and Cz) approximately corresponding to Fz, FCz, FC1, FC2 and Cz in the 10–20 system^33,34^ (Fig. 2).

## Supplementary information

Supplementary Information.

Supplementary Information.
