# Supplementary material for: Speech Intonation Induces Enhanced Face Perception in Infants
Source: Sci Rep. 2020 Feb 21;10:3225. doi: 10.1038/s41598-020-60074-7 (PMC7035392; doi:10.1038/s41598-020-60074-7)
Supplement: Supplementary file 1 — Supplementary Information. [file 41598_2020_60074_MOESM1_ESM.docx]

**Speech Intonation Induces Enhanced Face Perception in Infants**

Louah Sirri, Szilvia Linnert, Vincent Reid, & Eugenio Parise

**Supplementary Information**

Content

1. Additional Analysis: Infants Selection

2. Additional Analysis: Channels Selection

3. Additional Results: P400 Component

4. Additional Results: Comparison with Parise and Csibra

5. Figure S1: Regression Lines

**1. Additional Analysis: Infants Selection**

**1.1. Methods**

*Subjects*

In this analysis we included only those participants (Experiment 1: N=16; Experiment 2: N=16) contributing at a sufficient number of artifact free segments (at least 8) to both auditory and visual ERPs.

*EEG analysis*

The EEG analysis was performed the same way as described in the analysis section of the main paper.

**1.2. Results**

**Auditory ERPs**

*Experiment 1*

In the 200-400ms time window we did not find any significant main effect of Speech Type neither on the frontal electrodes nor on the temporal electrodes (all *p*s >.21). In the 600-800ms time window the effect of speech type was not significant on the frontal electrodes (*p* =.60), but it was significant on the temporal electrodes (*F*(1,15) = 5.90; *p* = .03; *η^2^_p_* = .28) with more negative amplitudes for IDS compared to ADS.

*Experiment 2*

In the 200-400ms time window the effect of Speech Type was not significant either on the frontal or on the temporal electrodes (all *p*s > .61). In the 600-800 ms time window the effect of speech type was significant both on the frontal (*t*(15) = -2.88, *p* =.01) and temporal electrodes (*F*(1,15) = 6.53; *p* = .02; *η^2^_p_* = .30) with larger amplitudes for IDS compared to ADS.

*Cross-experimental comparisons*

In the 200-400ms time window, the interaction Experiment x Speech Type was not significant on the frontal or on the temporal electrodes (all *p*s > .37). In the 600-800ms time window, the Experiment x Speech Type was not significant on the temporal electrodes *p* > .58) and it was marginally significant on the frontal electrodes (*F*(1,30) = 4.00; *p* = .06; *η^2^_p_* = .12).

**Visual ERPs**

*Experiment 1*

The main effect of Speech Type was not significant on the P1 or on Nc component (all *p*s > .11), but it was significant on the N290 (*F*(1,15) =14.01; *p* = .002; *η^2^_p_* = .48).

*Experiment 2*

The main effect of Speech Type was not significant on the P1 or on N290 component (all *p*s > .11), but it was significant on the Nc (*t*(15) = 3.66; *p* = .002)

*Cross-experimental comparisons*

The interaction Experiment x Speech Type was significant on the P1 and on the N290 component (P1: *F*(1,30) = 5.69; *p* = .02; *η^2^_p_* = .16; N290: *F*(1,30) = 6.37; *p* = .02; *η^2^_p_* = .18) and marginally significant on the Nc (*F*(1,30) = 3.99; *p* = .06; *η^2^_p_* = .12). The interaction on the P1 is due to change in the direction of IDS vs. ADS amplitude difference between Experiment 1 and 2 (Experiment 1: ADS>IDS; Experiment 2: IDS>ADS), while neither of those differences were significant themselves.

**1.3. Discussion**

When considering only those participants who contributed to both auditory and visual data, we found the same auditory and visual results we have got in the main analysis, with the only exception of the P1 result in Experiment 2. This suggests that the P1 result, that was originally not predicted, should be taken with caution. We conclude that all other results, particularly the main one on the N290, are solid and support our original hypothesis.

**2. Additional Analysis: Channels Selection**

**2.1. Methods**

*Subjects*

We used the same subjects’ selection as in the main text.

*EEG analysis*

In this analysis we included all posterior electrodes with identifiable P1 and N290 components. We averaged the visual ERPs over the left (channels: 58, 59, 64, 65, 66, 68, 69, 70, 71, 73, 74) and right (channels: 76, 82, 83, 84, 88, 89, 90, 91, 94, 95, 96) posterior areas. We conducted an ANOVA with Speech Type (IDS *vs.* ADS) and Hemisphere (left *vs.* right) as within-subject factors and analyzed the mean amplitudes for the P1 (100 to 200 ms) and the N290 (200 to 300 ms).

**2.2. Results**

*Experiment 1: Upright faces*

For the P1, the results showed no main effect of Speech Type nor Hemisphere (all *p*s > .45). There was, however, a significant main effect of Speech Type (F(1,18) =7.56; *p* =.01; *η^2^_p_*= .30) and Hemisphere (F(1,18) = 9.57; *p* =.006; *η^2^_p_*=.35) on the N290 component, with more negative amplitudes in the IDS relative to the ADS condition, and larger amplitudes over the right relative to the left hemisphere.

*Experiment 2: Inverted faces*

For the P1, the results showed a main effect of Speech Type (F(1,17) = 5.5; *p* =.03; ; *η^2^_p_* =.25) only, with more positive amplitudes in the IDS compared to the ADS condition. There was no significant main effect of Speech Type (F(1,17) = 1.18; *p* =.29; *η^2^_p_* = .07) nor Hemisphere (F(1,17) = 3.18; *p* =.09; *η^2^_p_* = .16) on the N290 component.

*Cross-experiment comparisons*

The interaction Experiment x Speech Type was significant for both the P1

(*F*(1,35) = 5.7; *p* = .02; *η^2^_p_* = .14), and the N290 (*F*(1,35) = 7.17; *p* = .01; *η^2^_p_* = .17).

**2.3. Discussion**

When including all electrodes with identifiable P1 and N290 components, for the visual ERPs we found the same pattern of results as in the main analysis. Notice that this extended electrodes’ selection overlaps with the scalp area analysed in (1) (we only excluded midline electrodes to maintain the factor Hemisphere). This shows that our main results are reliable, and are not due to ad hoc electrodes selection.

**3. Additional Results: P400 Component**

**3.1. Methods**

*Subjects*

We used the same subjects’ selection as in the main text.

*EEG analysis*

In this analysis we selected the same electrodes used in the main paper, that is a subset of those used in (1). We conducted an ANOVA with Speech Type (IDS *vs.* ADS) and Hemisphere (left *vs.* right) as within-subject factors and analyzed the mean amplitudes on the P400 (300 to 500 ms).

**3.2. Results**

*Experiment 1: Upright faces*

We found a significant main effect of Speech Type (F(1,18)=15.24; *p* =.001; *η^2^_p_*=.46) and Hemisphere (F(1,18)=7.64; *p* =.01; *η^2^_p_*=.30) on the P400 component, showing more positive amplitudes in the ADS relative to the IDS condition and over the left relative to the right hemisphere.

*Experiment 2: Inverted faces*

We did not find a significant main effect of Speech Type or Hemisphere (all *ps* >.17).

*Cross-experiment comparisons*

The interaction Speech Type by Experiment was significant (*F*(1,35) = 11.22; *p* = .002; *η^2^_p_* = .24).

**3.3. Discussion**

We found that the P400 results mirror those on the N290 component. We tentatively suggest that the P400 in this study likely reflect a carry-over effect from the N290.

**4. Additional Results: Comparison with Parise and Csibra**

**4.1. Methods**

*Subjects*

We used the same subjects’ selection as in the main text.

*EEG analysis*

We selected the same central electrodes (6, 7, 13, 30, 31, 37, 55, 80, 87, 105, 106, 112) used by Parise and Csibra (2). We conducted a series of t-tests on the auditory ERPs, with Speech Type (IDS *vs.* ADS) as within-subject factor and analyzed the mean amplitudes at 200 to 400 ms, 400 to 600 ms and 600 to 800 ms respectively.

**4.2. Results**

*Experiment 1: Upright faces*

We found no significant main effect of Speech Type at any time window (all *ps* >.31).

*Experiment 2: Inverted faces*

We found no significant main effect of Speech Type at any time window (all *ps* >.1).

*Cross-experiment comparisons*

We found no main effect of Speech Type (all *ps* >.55) or Speech Type by Experiment interaction (all *ps* >.13) at any time window.

**4.3. Discussion**

We did not replicate the results of (2). This can be due to a number of differences between the studies. Parise and Csibra used two disyllabic pseudo-words compliant to the Hungarian phonetic system (with the stress on the first syllable) and differing only in their intonation (the length was fully matched). As a consequence, the difference between IDS and ADS was strongly audible in the first half of the stimuli. Our stimuli differ more in their second half. Not to mention that the word “*hello*” we used should be already quite familiar to 4-month-olds.

In Parise and Csibra a face was continuously displayed on the screen, providing the infant with an obvious and plausible source of the speech.

Any of these differences could explain why we did not replicate the results of Parise and Csibra (2).

**References**

1. Farroni, T., Csibra, G., Simion, F., & Johnson, M. H. Eye contact detection in humans from birth. Proceedings of the National Academy of Sciences, **99**(14), 9602–9605 (2002).
2. Parise, E., & Csibra, G. Neural Responses to Multimodal Ostensive Signals in 5-Month-Old Infants. *PLoS ONE*, **8**(8), e72360 (2013).

**5. Figure S1: Regression Lines**


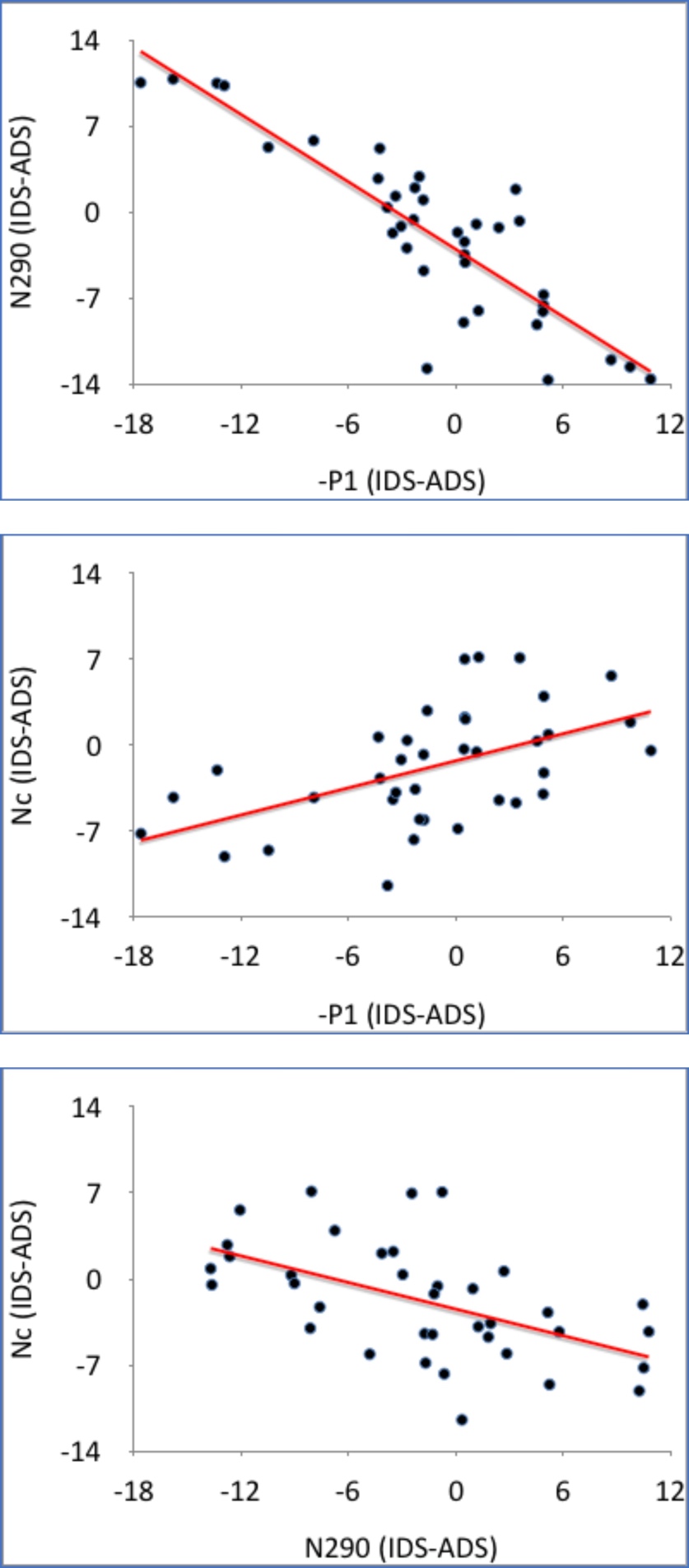


**Figure Legend**

**Figure S1.** Regression lines of pairwise correlations between -P1 and N290 (top panel), -P1 and Nc (middle panel), and N290 and Nc (bottom panel). All correlations are run on IDS-ADS amplitude differences.
